# Open fetal myelomeningocele repair with closure of a moderate-sized defect

**DOI:** 10.3171/2026.1.FOCVID25224

**Published:** 2026-04-01

**Authors:** Natalie Amaral-Nieves, Belinda Shao, Julie Monteagudo, Debra Watson-Smith, Petra Klinge, Francois I. Luks, Konstantina Svokos

**Affiliations:** Departments of 1 Neurosurgery and; 2 Pediatric Surgery, Brown University, Providence, Rhode Island

**Keywords:** prenatal repair, myelomeningocele, open

## Abstract

The authors present a case of open fetal myelomeningocele repair for a lumbosacral defect diagnosed at 19 weeks with ventriculomegaly and Chiari type II malformation. After multidisciplinary evaluation, the patient met MOMS (Management of Myelomeningocele Study)–based criteria and underwent fetal repair with placode dissection, dural and myofascial reconstruction, and AlloDerm-assisted skin closure. The postoperative course was stable, and premature rupture of membranes led to cesarean delivery at 35 weeks 4 days. The infant demonstrated spontaneous lower extremity movement and stable ventriculomegaly. At 10 months, he developed a dermoid cyst and tethered cord requiring surgery with good recovery. This case highlights the benefits and evolving long-term considerations of prenatal repair.

The video can be found here: https://stream.cadmore.media/r10.3171/2026.1.FOCVID25224

## Transcript

We present "Open fetal myelomeningocele repair with closure of a moderate-sized defect."

### 0:26 Case Presentation.

The patient’s mother is a 33-year-old female with history of 4 pregnancies and 3 full-term healthy deliveries who presents at 19 weeks of gestation with a male fetus. Patient does not take any medications aside from prenatal vitamins.

### 0:40 Preoperative Imaging.

Ultrasound revealed bilateral ventriculomegaly, evidenced by an enlarged left atrium, and a banana-shaped cerebellum suggestive of a small posterior fossa and Chiari II malformation. A lesion with splaying of the spinal elements was also visualized, consistent with an open neural tube defect.

MRI demonstrated moderately enlarged lateral ventricles and Sutton grade 2 hindbrain herniation confirming Chiari II malformation. A moderate sized 1.6 × 1.8–cm lumbosacral myelomeningocele was also noted from L4 to S5.

### 1:10 Perioperative Considerations.

The patient met criteria for open fetal myelomeningocele repair after multidisciplinary evaluation within the New England Fetal Treatment Program, aligned with the MOMS trial guidelines.^1,2^ Key fetal inclusion criteria included gestational age under 26 weeks, a lumbosacral lesion, the presence of a Chiari II malformation and moderate hydrocephalus consistent with an open defect, and a visible placode on fetal MRI. Maternal criteria included the absence of serious medical conditions, a BMI of 28.9, normal karyotype, posterior placenta, and no history of uterine surgery involving the active segment, uterine anomalies, or maternal hypertension.

Surgical risks were reviewed with the patient. Besides the known sequelae of cesarean delivery in future pregnancies, maternal risks related to the procedure include premature rupture of membranes, uterine wall thinning or dehiscence, and preterm labor. Fetal short-term risks include death, low birth weight, prematurity, and incomplete fetal wound healing with CSF leaks sometimes requiring postnatal surgical closure. Fetal repair risks include epidermoid cysts from residual epidermal elements occurring in 26%–30% of patients, often requiring reoperation with motor or bladder risk, and early symptomatic tethered cord from tissue scarring due to amniotic exposure.^3^

Our team consisted of maternal-fetal medicine, pediatric surgery, pediatric neurosurgery, obstetrical anesthesiology, and neonatology. Important perioperative considerations included antenatal steroids and magnesium sulfate for fetal pulmonary and neurological maturation, administration of tocolytics such as indomethacin and magnesium sulfate to reduce uterine contractility, maintenance of amniotic fluid volume and temperature with warmed Ringer’s lactate through an IV tube in the amniotic cavity, and anesthetic management aimed at optimal uterine relaxation while preserving placental perfusion. Essential equipment included sterile ultrasound, transuterine ultrasound for continuous fetal cardiac assessment by maternal-fetal medicine, dural substitutes such as DuraGen, an AlloDerm patch, a surgical microscope, and a neonatal incubator in case emergent delivery is necessary.

### 3:10 Patient Positioning and Exposure of Lesion.

In the operating room, the mother is placed supine with a roll under the right flank to minimize uterine compression of the inferior vena cava. The lithotomy position allows maternal-fetal medicine access for fetal cardiac evaluation during neurosurgical repair.

A low transverse laparotomy is performed. Under sterile ultrasound guidance, the placenta and fetal position are identified and marked to ensure the myelomeningocele lies beneath the planned uterine opening. The fetus may be gently repositioned by the OB-GYN as needed. A hysterotomy is initiated with two anchoring full-thickness sutures, followed by myometrial entry using electrocautery and placement of chromic purse-string sutures incorporating the membranes to avoid any disruption leading to bleeding. The incision is then extended to 6–8 cm using a Covidien Autosuture absorbable stapling device, which also helps maintain hemostasis.

Throughout the procedure, the uterus and membranes and the color of the amniotic fluid are carefully monitored visually and with ultrasound to monitor and control for any bleeding. No excess bleeding was encountered.

Through the uterine opening, the fetal back and the myelomeningocele defect are exposed. Intramuscular fentanyl and vecuronium are administered for fetal anesthesia and paralysis.

### 4:20 Primary Surgical Repair of Defect.

The neural placode appeared quite viable. In this case, to avoid undue manipulation, dissection with a 15 blade was carried out close to the dermal margins. Though excess arachnoid and microscopically visualized epithelialized elements were carefully trimmed with the microscissors, a fairly wider arachnoid plane was preserved, mainly to protect the delicate neural tissue and to ensure and allow sufficient tissue for neurulation in this case.

Following complete arachnoid dissection, the placode naturally repositions into the depth of the defect. In the present case, neurulation of the placode is performed by continuously closing the arachnoid membrane with a 5-0 PDS monofilament suture. This remains a topic of debate; some advocate for neurulation to reduce the risk of future retethering, while others avoid it due to potential injury to delicate neural tissue. To reconstitute a terminal thecal sac, the dural edges are mobilized and approximated with interrupted 5-0 PDS monofilament sutures. In cases where the dura is insufficient or friable, this step may be omitted and the available dural margins incorporated into the myofascial closure.

A variable amount of muscle can be elevated, but extensive dissection is often limited to avoid undue fetal manipulation and stress. In this case, the paraspinal fascia was available and used to support the previous approximation of the dural layer and carefully dissected with a 15-scalpel creating lateral ellipse cuts to allow tension-free coverage of the underlying dura and placode. Surgical site is consistently irrigated with normal saline.

A thin layer of DuraGen is then placed over the dural repair for reinforcement.

The fascial layer is then closed with a running 4-0 PDS monofilament suture to provide secure closure and optimal tissue support.

Skin edges are gently mobilized with Metzenbaum scissors to achieve a low-tension closure with well-vascularized tissue. The incision is closed with a running 4-0 PDS monofilament suture at the top and bottom, maintaining gentle approximation of the edges. When primary closure is not possible due to defect size or limited tissue mobilization, as in this case, an AlloDerm patch is used. The patch is tailored to the defect and secured with 4-0 PDS sutures, ensuring tension-free coverage and optimal healing.^4^ For more complex defects, myofascial or epidermal flaps may be employed.

### 6:42 Incisional Closure and Summary of Surgical Time.

The uterus is closed in three layers using 2-0 chromic sutures to restore strength and minimize postoperative bleeding. Following the first layer closure, an infusion of lactated Ringer’s solution and nafcillin is administered to optimize the amniotic fluid index. The abdominal wall and maternal skin are then closed in standard fashion.

The skin incision and exposure of the myelomeningocele took 1 hour and 3 minutes. The fetal myelomeningocele repair itself was completed in 33 minutes. Closure of the uterus and maternal abdominal incision required an additional 59 minutes.

### 7:14 Postoperative Course.

The patient is closely monitored in the PACU for 3 hours, with continuous assessment of contractions and fetal status, and remained stable throughout. She tolerated oral intake, ambulated without difficulty, and was discharged on postoperative day 4.

The fetus was safely delivered at 35 weeks and 4 days via cesarean section following premature rupture of membranes, weighing 2100 g. At birth, he demonstrated spontaneous movement of both lower extremities without spasticity. The fetal surgical incision was healing appropriately, with progressive epithelialization of the patch and no drainage or fluid collection. The patch underwent complete epithelization at 3 weeks, revealing a well-healed underlying incision.

Postpartum brain ultrasound and MRI demonstrated mild, stable ventriculomegaly and hindbrain herniation, and expected lumbosacral postoperative changes. He was discharged from the NICU on day 9.

In early outpatient follow-up, both mother and infant remained healthy. The child showed normal motor and urological developmental progress.

Approximately 1 year after fetal surgery, at 10 months of age, he began exhibiting signs of back discomfort and new urinary retention, for which urology initiated a catheterization regimen. MRI at that time revealed a fat-containing intradural mass with restricted diffusion extending from L3–4 to S2–3, most consistent with a dermoid/epidermoid tumor, as well as a low-lying conus at L3–4 and worsening holocord hydromyelia. Brain MRI showed stable ventriculomegaly and hindbrain herniation. We believe that, in this case, dissection a few millimeters lateral to the placode-arachnoid interface was a key factor contributing to the subsequent formation of this epidermoid cyst.

He subsequently underwent resection of the intradural mass and release of the tethered cord. Postoperative MRI demonstrated complete resection of the dermoid cyst and a reduction in syrinx size, with expected postoperative changes.

By the age of 3 years, he was ambulating with a walker and wheelchair for long distances and continued daily urinary catheterization. He remains shunt free with no symptoms of Chiari II malformation.

### 9:12 Discussion.

The MOMS trial showed fetal myelomeningocele repair lowers shunt need and improves motor and hindbrain outcomes, but later studies report mixed neurodevelopmental results.^5,6^ Some centers note equal or better postnatal outcomes, and long-term follow-up shows only partial persistence of benefit alongside rising concerns about early symptomatic tethered cord, declining ambulation, and frequent epidermoids.^7,8^ Minimally invasive fetoscopic and mini-hysterotomy approaches aim to reduce maternal morbidity. Fetoscopy was associated with fetal risks, while mini-hysterotomy lowers maternal risk without added fetal risk.^9,10^ Taken together, these findings suggest that the long-term durability of prenatal repair benefits is still being clarified.

Ultimately, maximizing outcomes from this procedure depends on thorough preoperative evaluation, optimizing precision in surgical techniques, and coordinated, long-term multidisciplinary care.

## Disclosures

The authors report no conflict of interest concerning the materials or methods used in this study or the findings specified in this publication.

## Author Contributions

Primary surgeon: Svokos. Assistant surgeon: Klinge, Luks. Editing and drafting the video and abstract: Amaral-Nieves, Klinge, Luks. Critically revising the work: Amaral-Nieves, Shao, Klinge, Luks, Svokos. Reviewed submitted version of the work: all authors. Approved the final version of the work on behalf of all authors: Amaral-Nieves. Supervision: Shao, Klinge, Luks, Svokos.

## Supplemental Information

### Patient Informed Consent

The necessary patient informed consent was obtained in this study.
